# Serum acute phase reactants hallmark healthy individuals at risk for acetaminophen-induced liver injury

**DOI:** 10.1186/gm493

**Published:** 2013-09-27

**Authors:** Jürgen Borlak, Bijon Chatterji, Kishor B Londhe, Paul B Watkins

**Affiliations:** 1 Centre for Pharmacology and Toxicology, Hannover Medical School, Carl-Neuberg-Straße 1, 30625, Hannover, Germany; 2 The Hamner Institutes for Health Sciences, 6 Davis Drive, Research Triangle Park, Box 12137, Durham, NC 27709, USA

## Abstract

**Background:**

Acetaminophen (APAP) is a commonly used analgesic. However, its use is associated with drug-induced liver injury (DILI). It is a prominent cause of acute liver failure, with APAP hepatotoxicity far exceeding other causes of acute liver failure in the United States. In order to improve its safe use this study aimed to identify individuals at risk for DILI prior to drug treatment by searching for non-genetic serum markers in healthy subjects susceptible to APAP-induced liver injury (AILI).

**Methods:**

Healthy volunteers (n = 36) received either placebo or acetaminophen at the maximum daily dose of 4 g for 7 days. Blood samples were taken prior to and after APAP treatment. Serum proteomic profiling was done by 2D SDS-PAGE and matrix-assisted laser desorption/ionization-time of flight-mass spectrometry. Additionally, the proteins C-reactive protein, haptoglobin and hemopexin were studied by quantitative immunoassays.

**Results:**

One-third of study subjects presented more than four-fold increased alanine transaminase activity to evidence liver injury, while serum proteomics informed on 20 proteins as significantly regulated. These function primarily in acute phase and immune response. Pre-treatment associations included C-reactive protein, haptoglobin isoforms and retinol binding protein being up to six-fold higher in AILI susceptible individuals, whereas alpha1-antitrypsin, serum amyloid A, kininogen and transtyretin were regulated by nearly five-fold in AILI responders. When compared with published findings for steatohepatitis and cases of hepatocellular, cholestatic and mixed DILI, 10 proteins were identified as uniquely associated with risk for AILI, including plasminogen. Notably, this zymogen facilitates macrophage chemotactic migration and inflammatory response as reported for plasminogen-deficient mice shown to be resistant to APAP hepatotoxicity. Finally, analysis of a publicly available database of gene expression profiles of cultures of human hepatocytes treated with drugs labeled as no- (n = 8), low- (n = 45) or most-DILI-concern (n = 39) confirmed regulation of the identified biomarkers to demonstrate utility in predicting risk for liver injury.

**Conclusions:**

The significant regulation of acute phase reactants points to an important link between AILI and the immune system. Monitoring of serum acute phase reactants prior to drug treatment may contribute to prevention and management of AILI, and may also be of utility for other drugs with known liver liabilities.

## Background

Drug-induced liver injury (DILI) is a major reason for drug failures in clinical trials, for withdrawal from the market or 'black box warnings' issued by the US Food and Drug Administration [1,2]. More than 1,000 drugs are suspected to cause liver injury in humans [3,4] and DILI accounts for more than 50% of acute liver failures (ALFs), with acetaminophen (APAP) hepatotoxicity far exceeding other causes of ALF in the United States [5]. It is perplexing that despite vigorous and extensive safety testing, animal studies fail to identify about 50% of drugs causing liver toxicity in clinical trials [6]. A major reason for drug withdrawal from the market or black box warnings is dose and treatment duration, particularly at prescribed daily doses of 100 mg or greater [7-9]. However, many drugs are safe at daily doses of 100 mg or higher with little or no risk of hepatotoxicity. A refined approach is therefore needed to predict risk for DILI. Importantly, with idiosyncratic DILI neither dose nor duration can be used to reliably predict hepatotoxicity. Idiosyncratic DILI is host dependent but not clearly dose related and frequently unrelated to the pharmacology of the drug; nonetheless, it is the most common reason for regulatory action and drug failures in clinical trials. Its pathologic mechanism is far from clear but may result from metabolic and or immune-mediated responses, with DILI histopathology revealing a broad spectrum of morphological presentations that are also common to other acute or chronic liver diseases.

Identifying individuals at risk for DILI prior to drug treatment would greatly improve drug safety and would have major implications for clinical practice. Although genetic associations with DILI susceptibility have been reported, none have yet been strong enough to be useful for managing DILI in the clinic. Indeed, in a most recent publication the DILI network reported the limited contribution of common genetic variants to risk for DILI for more than 200 drugs based on genome-wide association studies of 783 individuals who experienced significant liver injury [10].

Identifying non-genetic factors to predict individuals at risk for DILI prior to drug treatment would be a major breakthrough in its prevention. Furthermore, enabling outpatient monitoring of risk for DILI with a simple non-invasive test - measurement of biomarkers in either sputum or urine - would enable out-patients to monitor their risk for DILI and thus would help defeat the current limitations in post-marketing surveillance, with pharmacovigilance being frequently presumptive and primarily confined to signal detection.

This study aimed to identify non-genetic biomarkers to predict DILI susceptibility by serum proteomic profiling of healthy subjects prior to and after treatment with the maximum acceptable dose of APAP given in quarterly portions per day for 7 days. The serum proteins identified inform on the importance of acute phase reactants in predisposing individuals at risk to DILI.

## Methods

### Ethical statement

The study was approved by the ethics review board of the General Clinical Research Center at the University of North Carolina Hospitals, USA and was therefore performed in accordance with the ethical standards laid down in the 1964 Declaration of Helsinki and its later amendments. The approval was obtained by Dr Paul Watkins. Participants gave written informed consent before entering the study and were housed in clinical research facilities for the entire study.

### Study design

The original study protocol is described in detail in [11,12] and consisted of a two-center, randomized, single-blind (participants were blinded to treatment assignment), placebo-controlled, longitudinal design. Eligible participants were healthy men and women volunteers of non-childbearing potential aged between 18 and 45 years. Participants were considered to be healthy based on medical history, physical examination, electrocardiogram results and clinical laboratory measures (including negative urine drug screen, hepatitis B surface antigen and hepatitis C antibody results). No participants entered the study on concomitant medications. Race/ethnicity was self-reported; country of origin was not recorded.

Details regarding the study subjects are given in Additional file 1; each participant received either placebo or APAP. A total of n = 11 subjects on placebo (CB26-06 lot 2) and n = 25 receiving 1 g/qid APAP (4 g in total per day) were selected, of which n = 12 presented alanine transaminase (ALT) elevations of more than four-fold, on average, while n = 13 did not respond to this daily treatment for 7 days. No participant characteristics distinguished responders from non-responders, including urine metabolomic studies prior to dosing as recently reported [12].

### Preparation of serum samples

Blood serum samples from n = 36 participants were analyzed. After clotting for 30 minutes at room temperature, the blood was centrifuged at 2,000 rpm for 15 minutes. Hemolysis was not observed. The resultant supernatants were removed, frozen immediately in liquid nitrogen and stored at -80°C until further analysis. The protein concentration in serum was determined by the Bradford assay and ranged from 140 to 250 μg/μl.

### Serum proteomics

#### ***Removal of high abundant proteins***

Albumin and IgG were depleted in human serum using SpinTrap™ columns (GE Healthcare, Uppsala, Sweden). Columns are prepacked with a mixture of anti-HSA Sepharose High Performance and Protein G Sepharose High Performance. The mixed media consists of 34 μm high cross-linked agarose beads with covalently immobilized affinity ligands. Depletion columns were applied according to the manufacturer’s instructions. A binding buffer consisting of 20 mM sodium phosphate, 0.15 M sodium chloride, pH 7.4 was used. Undiluted human serum (50 μl) was diluted with binding buffer to a final volume of 100 μl before application to the column. The protein concentration in depleted serum determined by the Bradford test ranged from 3.2 to 9.2 μg/μl. Samples were purified thereafter using the ReadyPrep™ 2D Cleanup kit (BioRad, Hercules, CA, USA) to improve isoelectric focusing (IEF) resolution by removing salts, contaminants, detergents, lipids and other compounds.

#### ***One- and two-dimensional SDS-PAGE***

##### 

**First dimension** Serum proteins were separated by IEF and precast immobilized pH gradient (IPG) strips of pH 3 to 10 (non-linear gradient; 170 × 3 × 0.5 mm, BioRad) and pH 4 to 7 (linear gradient; 170 × 3 × 0.5 mm, BioRad) were used. Each sample was analyzed in duplicate. We diluted 300 μg in a lysis buffer containing 2 mol/l thiourea, 5 mol/l urea, 40 mmol/l Tris, 4% CHAPS, 0.5% BioLyte 3-10 (BioRad), and 100 mmol/l dithiothreitol, resulting in a total volume of 300 μl per strip. Focused IPG strips were rehydrated at 50 V for 12 h. IEF was performed at 20°C with a maximum voltage of 10 kV and a maximum current of 50 μA per strip. After IEF, IPG strips were equilibrated in 10 ml reducing buffer (2% dithiothreitol in 10 ml equilibration buffer containing 6 mol/l urea, 30% glycerine, 2% SDS, 0.05 mol/l Tris-HCl, pH 8.8 and 0.5% bromophenol blue) for 15 minutes, followed by equilibration in 10 ml alkylation buffer (4% iodoacetamide and 0.5% bromophenol blue in 10 ml equilibration buffer) for 15 minutes.

##### 

**Second dimension** SDS-PAGE was performed in a Protean-plus Dodeca™ Cell (BioRad) using self-cast polyacrylamide gels (200 × 205 × 1.5 mm; 12%T). Gels were run in parallel in 0.025 mol/l Tris/0.192 mol/l glycine/0.1% SDS cooled externally to 10°C with a constant voltage of 70 V for approximately 16 h, followed by 200 V until the bromophenol blue dye reached the bottom of the gel. Unstained protein standards (BioRad) were used for calibration of M_r_ and pI.

#### ***Staining***

Two-dimensional gels were fixed overnight in 500 ml 30% ethanol/2% phosphoric acid and washed twice for 30 minutes each in 500 ml 2% phosphoric acid. Equilibration was done in 500 ml 2% phosphoric acid/18% ethanol/15% ammonium sulfate for 30 minutes thereafter. Colloidal Coomassie Brilliant Blue (CBB) staining of proteins was performed by addition of 5 ml staining solution (2% CBB G250, Roth, Karlsruhe, Germany) to 500 ml of equilibration solution. Gels were stained for 72 h and washed once with 500 ml deionized water for 5 minutes thereafter.

#### ***Imaging***

Gels were scanned as TIFF images with the 16-bit Expression 10000 XL™ (Epson, Nagano, Japan) operating in transmitted light mode. PDQuest™ software (version 8.0.1.55, BioRad) was used for detection of spots, quantification and comparison of two-dimensional protein profiles. Background and vertical streaks were removed from each gel image; spots were digitized by Gaussian fit. To quantify protein spots, a match set of all gels was made and the absorbance of individual protein spots from two-dimensional gels was measured. Total density in the gel image was used for normalization of spots. In this method, the raw quantity of each spot in a member gel is divided by the total intensity value of all the pixels in the image. This model assumes that the total density of an image (that is, background density plus spot intensity) will be relatively consistent from gel to gel. Student’s *t*-test and Mann-Whitney signed-rank test were used for analysis of protein expression. A probability of *P* < 0.05 was considered statistically significant. Differentially regulated spots were excised and transferred to 96-well microtiter plates (Thermo, Waltham, MA, USA) by the EXQuest™ spot cutter (BioRad) for further identification by mass spectrometry.

#### ***Matrix-assisted laser desorption/ionization-time of flight-mass spectrometry***

Each of the CBB-stained gel plugs was washed twice with 15 μl ammonium hydrogencarbonate solution (100 mmol/l) and then dehydrated twice with 15 μl acetonitrile. Proteins were digested with a total of 80 ng trypsin (sequencing grade, Promega, Madison, WI, USA) per gel plug at 37°C for 4 h. Resulting peptides were extracted with 1% trifluoroacetic acid in an ultrasonic bath (Sonorex, Super RK 514 BH, Bandelin, Berlin, Germany) for 10 minutes.

The peptide extracts (2 μl) were manually spotted onto a 600 μm/384 well AnchorChip™ sample target (Bruker Daltonics, Bremen, Germany) and dried at room temperature. The matrix-assisted desorption/ionization (MALDI) target was covered with a thin layer of alpha-cyano-4-hydroxycinnamic acid matrix dissolved in 97% acetone/3% trifluoroacetic acid 0.1% to saturation. To enable tandem mass spectrometry measurement, matrix re-crystallization was performed with 0.4 μl of 60% ethanol/30% acetone/10% trifluoroacetic acid 1%. MALDI mass spectra were recorded using an Ultraflex II time of flight (TOF)/TOF mass spectrometer (Bruker Daltonics) equipped with a 384-sample scout source. An external peptide calibration standard containing the following fragments was used to calibrate the instrument: angiotensin II ([M + H]^+^ 1046.54); angiotensin I ([M + H]^+^ 1296.68); substance P ([M + H]^+^ 1347.74); bombesin ([M + H]^+^ 1619.82); ACTH clip 1-17 ([M + H]^+^ 2093.09); ACTH clip 18-39 ([M + H]^+^ 2465.20); somatostatin 28 ([M + H]^+^ 1347.47) (Bruker Daltonics). Mass spectra were acquired in an automatic mode using the AutoXecute™ module of the FlexControl™ software (version 2.4, Bruker Daltonics) and using the three most abundant peptide signals of the corresponding peptide mass fingerprint and peptide fragmentation fingerprint spectra. Spectra were analyzed using the FlexAnalysis™ software (version 2.4, Bruker Daltonics). The Swiss-Prot database (download 2005) employing the MASCOT program (version 2.0, in-house server, Matrix Science, Boston, MA, USA) was used to search for peptide masses to identify proteins. Database searches were performed taking into account carbamidomethyl modification of cysteines and possible oxidation of methionine. One missed cleavage was allowed. A mass inaccuracy of ≤100 ppm was allowed for the peptide mass fingerprint. For the peptide fragmentation fingerprint, a mass inaccuracy of ≤70 ppm was allowed for both peptide masses and their fragments. Identified proteins were sent to the Proteinscape™ database (version 1.3, Protagen, Dortmund, Germany) and checked individually for further consideration.

#### ***Clinical chemistry measurements of C-reactive protein, haptoglobin and hemopexin***

Serum C-reactive protein (CRP), haptoglobin and hemopexin concentrations were determined by quantitative immunoassays using standard operating procedures of the Institute of Clinical Chemistry at the University of Regensburg, Germany.

#### ***Sputum C-reactive protein measurements***

Samples were collected from healthy volunteers at the Biomedical Research Center of Uppsala University, Sweden (Laboratory of Dr Lars Baltzer). The volunteers had rinsed their mouth with water 10 minutes before collection and had not eaten within 1 h before sample collection. The samples were immediately frozen and then stored at -20°C.

Saliva samples were thawed at room temperature and centrifuged prior to analysis. A standard curve with human CRP spiked in 0.05% bovine serum albumin was prepared at a concentration range of 0 to 5,000 pg/ml, which is within the range of interest for CRP measurements in saliva (Additional file 2: Figure S1A; samples in triplicates). Here the level of the blank samples (no CRP) was 0.065. Measurement of the samples from healthy volunteers resulted in a response of 0.070 and 0.075, respectively (triplicates). In addition, a standard curve with human CRP, 5,000 to 0 pg/ml spiked in saliva (4× diluted), was also prepared (Additional file 2: Figure S1B; samples in triplicates).

### Human hepatocyte studies

The publicly available Japanese Toxicogenomics Project data set was used [13] and analyzed with respect to serum DILI biomarker candidates identified in the present study.

### Statistical analysis

Statistical analysis was done with GraphPad Version 5.0 and SPSS software version 17 using a non-parametric testing strategy. Furthermore, the MAS5 algorithm for generating expression summaries and the Bioconductor R package were used for the analysis of the publicly available database (see above human hepatocyte studies and [13]). For individual genes the ratio of control versus treatment was determined. ANOVA was done using the aov function in the stats package of R, and box plots were drawn with R. Statistically significant changes are marked with asterisks in the figures.

## Results

### Alanine transaminase activity in response to APAP treatment

**Figure 1:**
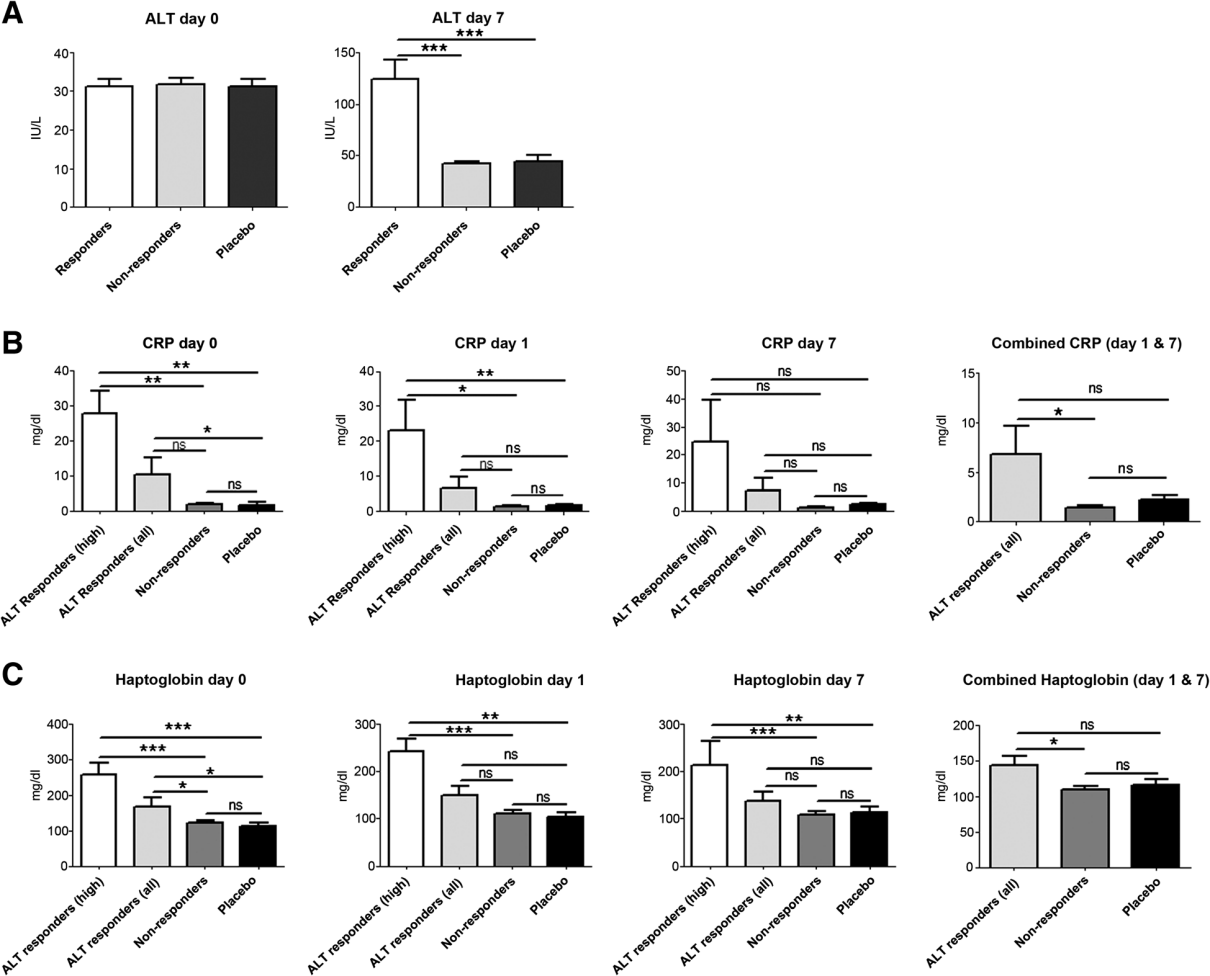
**Serum alanine transaminase activity, CRP and haptoglobin prior to and after treatment of healthy subjects receiving 4 g of acetaminophen daily for 7 days. (A)** Serum alanine transaminase activity (ALT) prior to and after APAP treatment of healthy subjects for 7 days. Depicted are ALT activities of DILI-responders versus non-responders receiving the same APAP dose. ALT activity in placebo-treated healthy volunteers is also shown. Note that the ALT activity was unchanged between pre-treatment and day 1 measurements. **(B)** CRP serum concentration of DILI responders with either high CRP (n = 3 healthy individuals) or all DILI responders, DILI non-responders and placebo-treated healthy subjects at baseline, day 1 and day 7 of APAP treatment. CRP measurements combined for day 1 and day 7 are also shown. **(C)** Haptoglobin serum concentration of DILI responders with either high haptoglobin (n = 3 healthy individuals) or all DILI responders, DILI non-responders and placebo-treated healthy subjects at baseline (day =0), day 1 and day 7 of APAP treatment. Haptoglobin measurements combined for day 1 and day 7 are also shown. ***P < 0.001; **P < 0.01; *P < 0.050; ns, not significant.

The mean ALT activity of healthy volunteers prior to APAP and/or placebo treatment is depicted in Figure 1A and was within normal range (median = 30) amongst all study participants. Doses of 4 g APAP daily for 7 days caused a ≥4-fold increases in ALT activity in about one-third of study participants (Figure 1A; median = 120). The increase in ALT was transient and returned to normal shortly after drug removal and none of the participating study subjects experienced severe liver injury. Study subjects were divided into 'DILI responders’ with significantly increased ALT and 'DILI non-responders’ in whom ALT activity remained normal after repeated APAP treatment.

### Differentially expressed proteins in native serum of DILI-responders prior to APAP treatment

**Figure 2:**
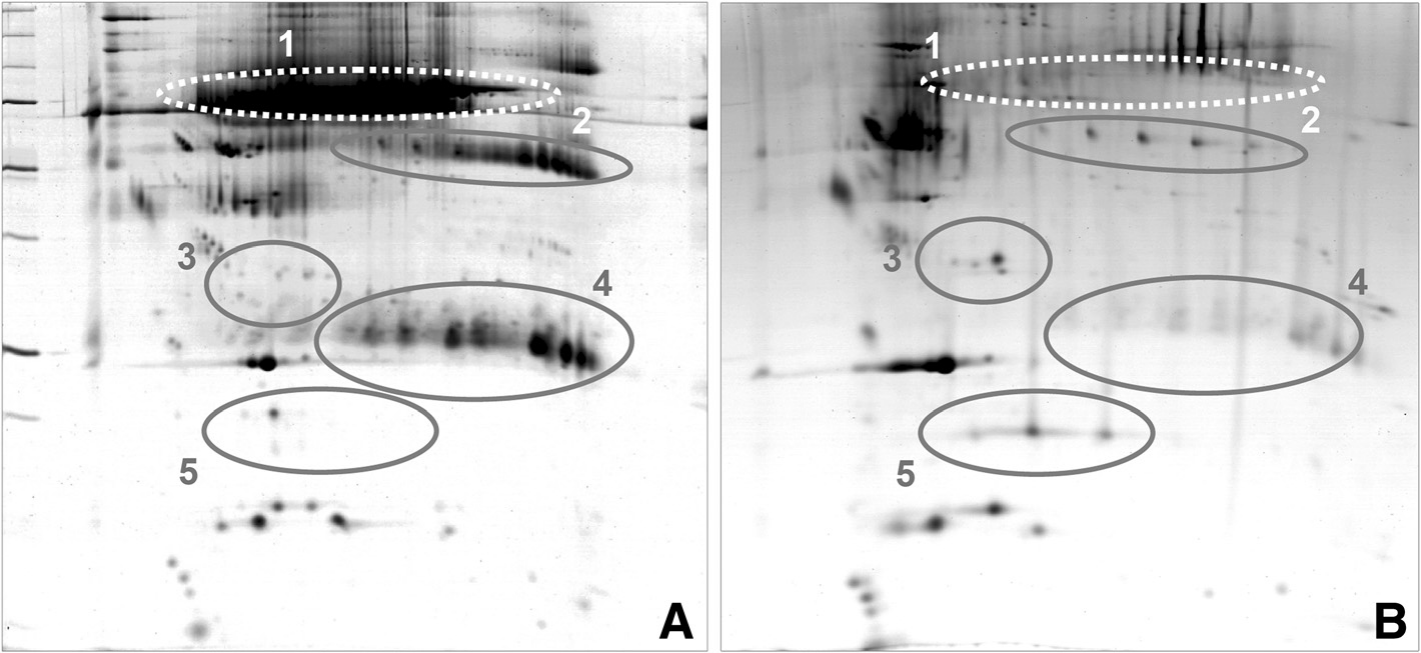
**Two-dimensional gel electrophoresis of serum proteins of healthy subjects receiving 4 g of acetaminophen daily for 7 days. (A)** Native serum of healthy volunteers or **(B)** after immunodepletion of high abundant proteins, such as serum albumin (1) and immunoglobulin G (4). An improved resolution of apolipoprotein H (2) and apolipoprotein E (3) and haptoglobin (5) was achieved in depleted sera.

A thiourea-containing lysis buffer was used to extract proteins from serum as recently described [14]. As high-abundant serum proteins such as albumin and immunoglobulins may interfere with the separation of complex protein mixtures, extracts from native and albumin-depleted serum were prepared and separated by two-dimensional electrophoresis (Figure 2). Two-dimensional gel electrophoresis was carried out at pH 3 to 10 and/or 4 to 7 and subsequently visualized with the CCB stain. A detailed description of the workflow, including protein extraction, two-dimensional gel electrophoresis, image processing, spot-cutting, trypsin in-gel digestion and MALDI-TOF/TOF mass spectrometry of proteins, is given in Additional file 3.

**Figure 3:**
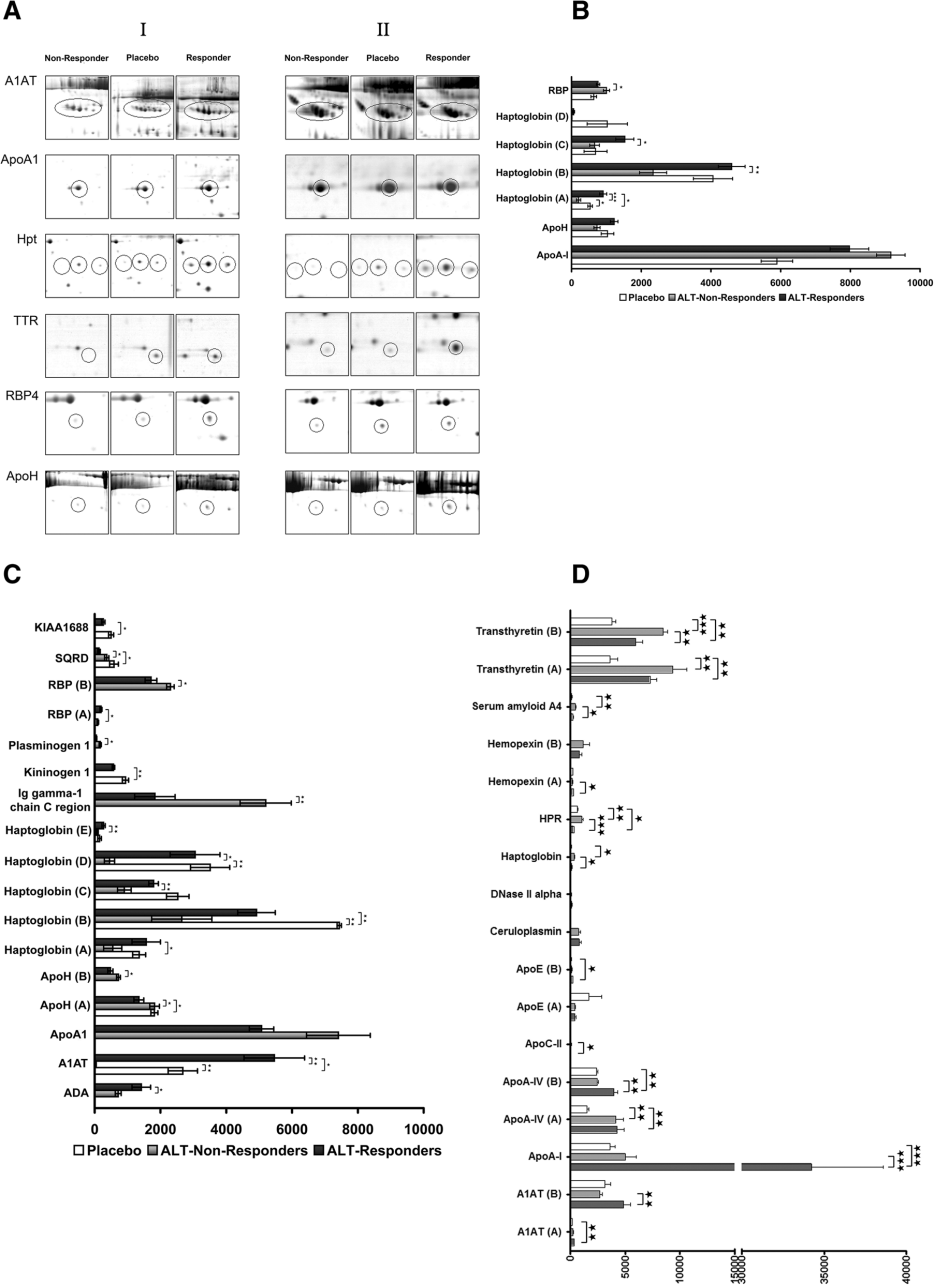
**Sections of two-dimensional electrophoresis gels showing regulated serum proteins from healthy volunteers. (A)** ALT responders, ALT non-responders, placebo prior to APAP or placebo treatment (day 0, left panel, I) and after daily APAP treatment for 7 days (right panel, II). A1AT, alpha-1-antiproteinase; ApoA1, apolipoprotein A-I; Hpt, haptoglobin; TTR, transthyretin; RBP4, retinol binding protein 4; ApoH, apolipoprotein H. **(B)** Results from densitometric scans of two-dimensional electrophoresis images of serum proteins regulated in DILI responders, non-responders and placebo-treated healthy volunteers prior to APAP treatment. **(C)** Results from densitometric scans of two-dimensional electrophoresis images of serum proteins regulated in DILI responders, non-responders and placebo-treated healthy volunteers after APAP treatment for 7 days. **(D)** Results from densitometric scans of two-dimensional electrophoresis images of serum proteins after immunodepletion of high abundance proteins in DILI responders, non-responders and placebo-treated healthy volunteers after APAP treatment for 7 days. ***P < 0.001; **P < 0.01; *P < 0.05.

Six proteins were identified as differentially expressed in DILI responders prior to APAP treatment (Figure 3A). However, only haptoglobin isoforms, retinol binding protein (RBP)4 and CRP (Figures 1B and 3B) reached statistical significance. Remarkably, RBP expression was higher in non-responders while CRP and haptoglobin expression was higher in DILI responders. The serum concentrations of CRP, haptoglobin and hemopexin were additionally analyzed by immunoassay. The baseline CRP serum concentrations in 3 out of 12 ALT responders was 27 mg/dl and thus nearly three-fold above ULN (Figure 1B), while the overall mean for all ALT responders was close to the upper limit of normal, that is, 10 mg/l When CRP baseline measurements for all DILI responders were compared to the placebo arm of the study a likewise statistically significant six-fold increase was determined. However, CRP serum concentration did not differ after single and repeated APAP treatment. Thus, CRP measurements were combined within study groups. A statistically significant difference was determined for the comparison of all DILI responders versus non-responders (Figure 1B); CRP serum concentrations and ALT activity appeared to be correlated (Additional file 4). Additional file 2 informs on CRP measurements in saliva to demonstrate its facile monitoring by non-invasive means.

Haptoglobin serum concentrations were also determined by immunoassay but the employed assay can not distinguish between various isoforms. As depicted in Figure 1C, the haptoglobin baseline was significantly higher in DILI responders when compared with non-responders or study subjects receiving placebo. Of note, two individuals with slightly above normal CRP baseline values also presented higher haptoglobin serum concentrations. Removing these individuals from the data analysis did not change the statistical significance. No obvious treatment effect on haptoglobin serum concentration was observed. Thus, haptoglobin measurements for day 1 and 7 were combined; a statistically significant difference for all DILI responders compared with non-responders was determined.

Based on two-dimensional electrophoresis mass spectrometry, up-regulation of other serum acute phase reactant and inflammation markers was identified, including serum amyloid A4, alpha-1 antiproteinase (A1AT), haptoglobin isoforms as well as kininogen. Conversely, immunoglobulin gamma-1 chain C region (IGHG1) and RBP4 were reduced in expression after APAP treatment for 7 days (Figure 3C). Likewise, significant up-regulation of adenosine deaminase (ADA) but reduced expression of apolipoprotein (Apo)A-I, ApoH, plasminogen and sulfidequinine oxidoreductase were observed in DILI responders after repeated APAP treatment. Prominent examples of regulated proteins are depicted in Figure 3A.

### Differentially expressed proteins in albumin and immunoglobulin-depleted serum of DILI responders after APAP treatment

To further search for regulated proteins, the high abundance serum proteins albumin and immunoglobulin were removed by the use of the SpinTrap column technology. Efficient immunodepletion of high abundant proteins was achieved as determined by two-dimensional gel electrophoresis (Figure 2). Similar to un-fractionated sera of APAP-treated healthy volunteers, A1AT was significantly up-regulated while haptoglobin, serum amyloid A4 and transthyretin were down-regulated. Moreover, induced expression of ApoA1, A4, C-II and E and, after removal of albumin, regulation of ceruloplasmin, deoxyribonuclease-2-alpha and hemopexin were observed in APAP-treated healthy volunteers.

### Correlation between ALT activities and expression of acute phase reactants prior to and after APAP treatment

**Figure 4:**
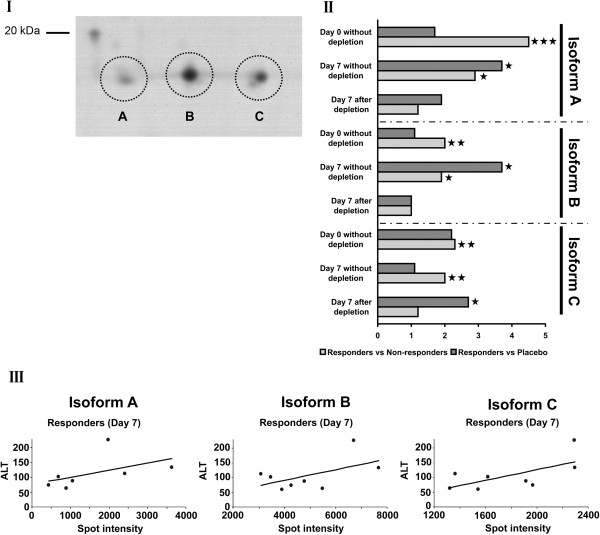
**Isoforms of haptoglobin identified as regulated in healthy volunteers prior to APAP treatment. (I)** Two-dimensional gel electrophoresis showing three major isoforms of haptoglobin. **(II)** Results of densitometric scans of two-dimensional electrophoresis images of serum proteins regulated in DILI responders, non-responders and placebo-treated healthy volunteers prior to and after APAP treatment for 7 days. **(III)** Correlation of serum ALT activities and haptoglobin isoforms in DILI responders after APAP treatment for 7 days. ***P < 0.001; **P < 0.01; *P < 0.05.

Amongst the acute phase reactants, haptoglobin isoforms were chosen and found to correlate with ALT activities of individual study subjects. The relative spot intensities of three major isoforms, denoted as spots A, B and C in Figure 4, from individual DILI responders correlated with ALT activity in response to APAP treatment. Similar correlations were observed with other acute phase reactants (data not shown).

**Figure 5:**
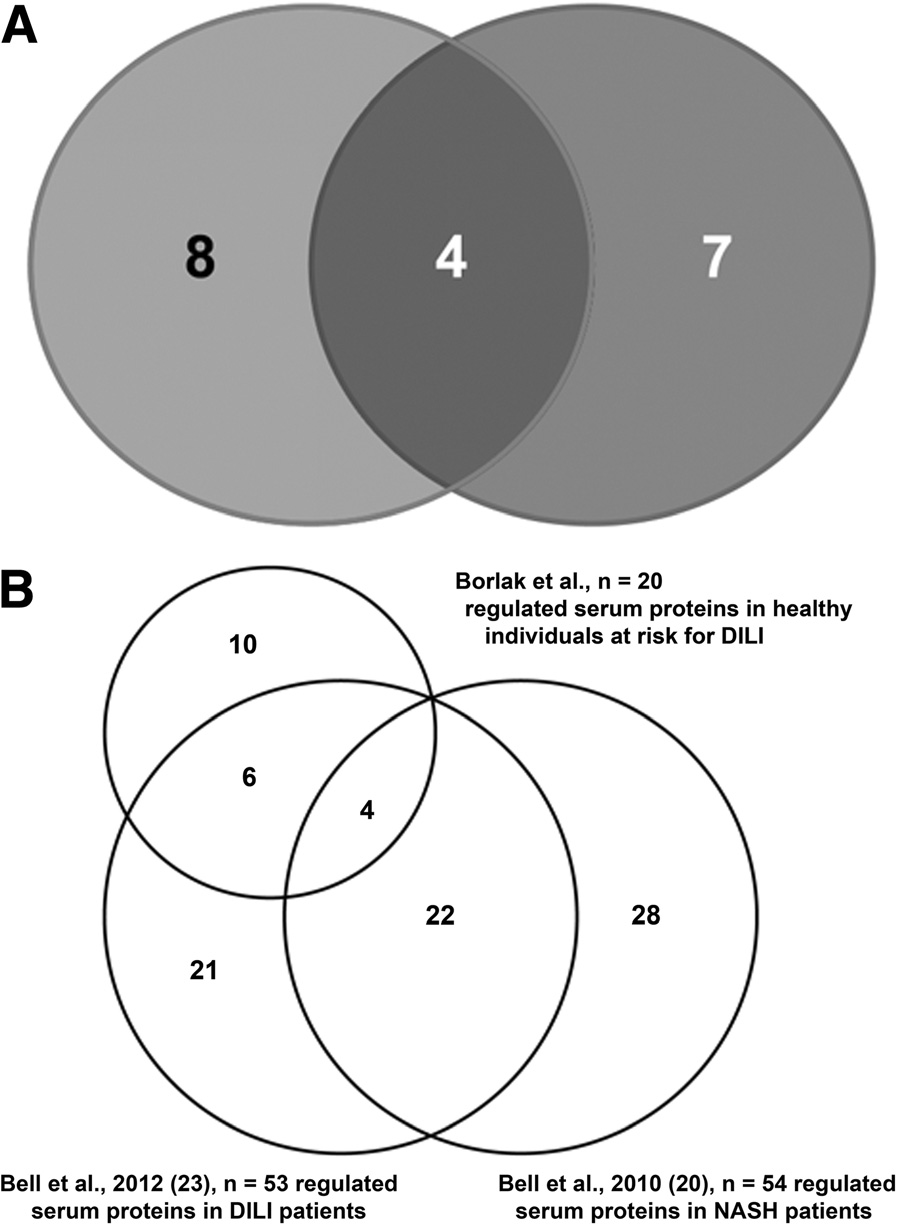
**Regulation of serum proteins in DILI and other liver diseases. (A)** Venn diagram of regulated proteins in native (left circle) and albumin-depleted (right circle) sera of DILI responders. Four proteins were common amongst native and albumin- and IgG-depleted sera. **(B)** Venn diagram of the findings of the present study with reports for patients diagnosed with mild, moderate and severe DILI and/or non-alcoholic steatohepatitis (NASH) [20,23].

Overall, 20 proteins were identified as regulated in sera of healthy volunteers treated with APAP. Of these, four were regulated in common in native and albumin-depleted sera - A1AT, ApoA-I, transthyretin and haptoglobin. In native sera of APAP-treated healthy volunteers plasminogen, RBP4, beta-2-glycoprotein 1 (ApoH), ADA, kininogen-1, IGHG1, pre-mRNA-splicing factor SYF1 and sulfide quinone oxidoreductase were regulated. Removal of albumin identified either regulated or de novo expressed serum proteins, that is, the ApoA-IV, C-II and E as well as ceruloplasmin, deoxyribonuclease-2-alpha and hemopexin. These findings document the importance of comparative proteomic studies (Figure 5A). Additionally, the results of the present study were compared with published data of serum proteomic profiling in patients with non-alcoholic steatohepatitis or DILI. This revealed 10 proteins as uniquely associated with APAP-induced liver injury (AILI) prior or in response to drug treatment in healthy subjects (Figure 5B; Additional file 5).

### Human hepatocyte studies

**Figure 6:**
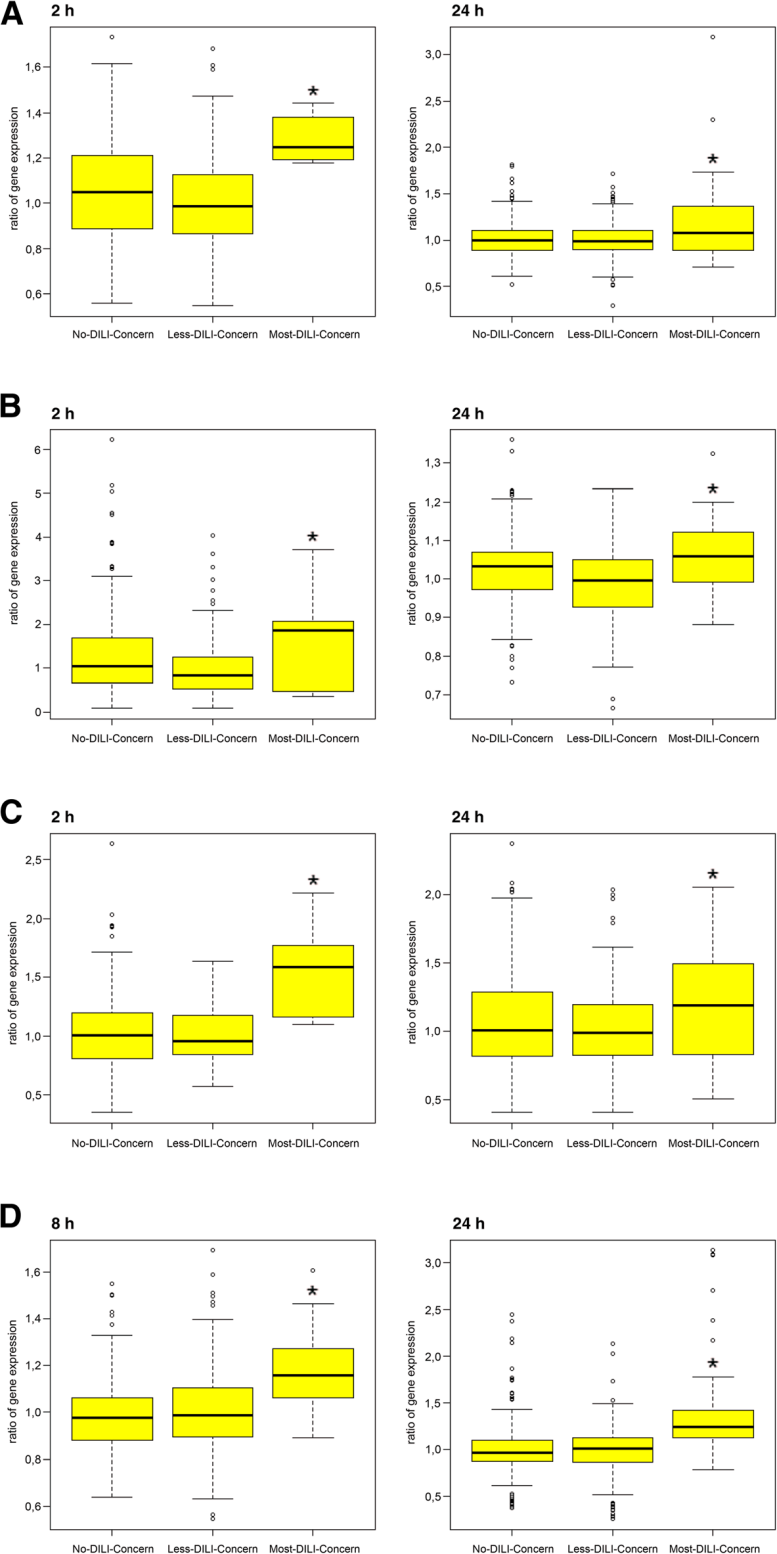
**Box plots of gene expression changes after treatment of cultures of human hepatocytes with drugs labeled as no-, less- or most-DILI-concern. (A)** Serum amyloid. **(B)** Sulfide quinone oxidoreductase. **(C)** Apolipoprotein C2. **(D)** Ceruloplasmin. Box blots marked with a star are statistically significant at *P* < 0.05. Cultures of human hepatocytes were treated with drugs labeled as either no- (n = 8), low- (n = 45) or most-DILI-concern (n = 39) for 2 and 24 h, respectively. A full list of drugs used is given in Additional file 6.

Publicly available data were retrieved [13] and analyzed for drugs labeled as either no- (n = 8), low- (n = 45) or most-DILI-concern (n = 39). This confirmed regulation of newly identified serum biomarkers of DILI susceptibility at the transcript level for a wide range of idiosyncratic agents and suggests a role of acute phase reactants in different mechanisms of liver injury (Figure 6 and Additional file 6).

## Discussion

AILI is a prominent cause of ALF in the US; it can primarily be attributed to unintentional as well as intentional ingestion of above therapeutic doses [15]. However, 7% of acute liver failures are at therapeutic doses, as reported by the acute liver failure group, who determined the incidence, risk factors, and outcomes of APAP-induced ALF at 22 tertiary care centers in the United States. As APAP serum concentrations in cases of ALF at therapeutic APAP doses were similar to those in overdose patients and the median APAP concentration differed by only a factor of two (14. 5 μg/dL versus 31 μg/dL), one might possibly suggest self-reported doses and time of ingestion to be unreliable. The seminal study of Larson et al. [15] reported a dramatic increase in APAP-induced ALF particularly in patients with chronic pain and co-medications (narcotics and/or depressants) as well as alcohol abuse. Thus, identifying biomarkers that would predict individuals at risk for AILI prior to drug treatment would greatly improve drug safety. For this purpose the serum proteome of healthy subjects was analyzed prior to and after treatment with the maximum acceptable dose for APAP given in quarterly portions per day for 7 days. This identified important pre-treatment serum protein associations in individuals that subsequently developed liver injury upon drug treatment.

### Regulation of acute phase proteins in AILI responders

A significant difference in expression of serum markers of inflammation was observed in AILI responders versus non-responders. Of the various positive and negative acute phase reactants, CRP, haptoglobin, ApoA-I, beta-2 glycoprotein 1 (ApoH) and RBP are important pre-treatment associations. The mean CRP in 3 out of 12 DILI susceptible subjects was nearly 3-fold higher than the normal upper limit, possibly indicating a mild pre-existing inflammation even though active inflammation of the liver would be associated with CRP levels well above 100 mg/L. Nonetheless, all subjects entering the study were vigorously tested and ALT as well as all other laboratory parameters were within normal ranges. While pre-dose CRP serum concentrations were significantly elevated in ALT responders, its expression was basically unchanged after 7 days of treatment. Notably, CRP serum concentrations and peak ALT measurements appear to correlate (Additional file 4). It is of considerable importance that CRP stimulates phagocytosis of Kupffer cells but decreases their TNF production in a complex manner in which various Fc receptors are involved [16].

A further biomarker for DILI susceptibility is haptoglobin. This positive acute phase reactant functions as a protective anti-oxidant [17] and plays a role in the reticuloendothelial system. This system is composed of monocytes and macrophages and is part of the immune system to remove cell debris as observed in cytolitic hepatitis. Haptoglobin binds hemoglobin and plays a crucial role in heme iron recovery to prevent its loss and to protect kidneys from damage by renal iron loading [18]. Prior to APAP treatment, haptoglobin isoforms with different pI but similar M_r_ (<20 kDa) were significantly (*P* < 0.01) up-regulated in ALT responders (Figures 3C and 4). Of note, the isoforms remained significantly up-regulated after repeated APAP treatment for 7 days. Diverse functions have been attributed to this tetrameric glycoprotein, with recent evidence pointing to a function as a chemo-attractant for macrophages [19].

Haptoglobin deficiency is associated with attenuation of hepatosteatosis and impairment of glucose homeostasis, suggesting this protein has a wider role in liver injury. Because of haptoglobin’s role in iron metabolism, the expression of hemopexin was studied as well but remained unchanged in all of the study groups investigated (Additional file 7).

Additional markers of inflammation were significantly regulated and included the acute phase reactant A1AT in native and albumin-depleted sera of ALT responders. This serpin is mainly produced by the liver and irreversibly inhibits trypsin, chymotrypsin and plasminogen activator. In DILI patients the serum concentration of alpha-1-antitrypsin and activity of the transaminases ALT and aspartate aminotransferase (AST) are significantly correlated [20]; however, A1AT serum concentrations are reduced in ALF [21]. Infantile liver cirrhosis is linked to carriers of the homozygous allele Z or M-Malton of the A1AT gene [22] while the Pittsburgh variant causes hemorrhagic diathesis.

Another major acute phase reactant significantly up-regulated in AILI susceptible subjects is serum amyloid A (SAA). During an acute phase response this protein is rapidly synthesized by the liver; its expression is regulated by IL-1, IL-6 and TNFα. SAA is an apolipoprotein (apoSAA) associated with high-density lipoprotein and together with CRP are sensitive markers for inflammation. In DILI patients SAA proteins are significantly regulated; a positive correlation between alkaline phosphatase activity and expression of serum amyloid A2 (isoform a) has been reported [23].

Conversely, APAP treatment caused significant down-regulation of the negative acute phase reactant transthyretin in AILI responders. This triiodothyroxine binding protein is referred to as pre-albumin and is significantly regulated in inflammation. Transthyretin interacts with RBP and was reported as significantly regulated in DILI [23] and non-alcoholic fatty liver disease (NAFLD) patients of different types [20]. It was also shown to be initially repressed but subsequently up-regulated by more than six-fold in sera of APAP-treated rats after 6 and 24 h, respectively [24].

About 40% of plasma transthyretin circulates as a complex with RBP. This protein is another negative acute phase reactant and a member of the lipocalin family; the complex stabilizes the binding of retinol to RBP to decrease its glomerular filtration and renal catabolism. Importantly, RBP was significantly down-regulated in ALT responders amongst APAP-treated healthy volunteers; a similar two-fold down-regulation was reported for APAP-treated rats [24]. Independent studies with DILI and NAFLD patients observed similar regulation of RBP [20,23].

A further acute phase reactant regulated in albumin-depleted sera of ALT responders is ceruloplasmin. This multicopper oxidase plays an important role in oxygen detoxification and is up-regulated during inflammation and hyperoxia [25]. The enzyme oxidizes Fe2+ to Fe3+ without release of radical oxygen species. It is involved in iron transport across the cell membrane. Ceruloplasmin is synthesized by the liver and secreted into plasma by hepatic stellate cells as well as Kupffer cells [26]. In Morbus Wilson patients ceruloplasmin serum levels are decreased.

### Drug-induced liver inflammation and immune response

Of particular interest is ADA, which is expressed in all tissues and in large amounts of T lymphocytes. Expression of this enzyme was significantly up-regulated in native serum of ALT responders after 7 days of APAP treatment. The release of ADA into serum documents cellular damage in response to APAP treatment. An important function of serum ADA is the conversion of adenosine to the nucleoside inosine, which is unable to bind to adenosine receptors, thereby influencing neutrophil degranulation, blood flow and oxygen consumption. It is tempting to speculate that increased serum ADA results in the production of anti-inflammatory molecules similar to those seen with non-selective adenosine receptor antagonists, that is, xanthine derivatives. Lack of ADA causes immunodeficiency and knockout mice die perinatally due to severe liver cell degeneration [27,28]. ADA deficiency is also associated with hyperbilirubinemia and hepatitis but can be resolved by ADA replacement therapy [29]. In acute infective hepatitis serum ADA levels increase; a positive correlation between serum ADA and total bilirubin was reported [30]. This positive correlation may suggest bilirubin functions as an anti-oxidant [31].

In DILI responders and after repeated APAP treatment IGHG1 was significantly reduced. Importantly, immunoallergic DILI is associated with idiosyncratic liver injury. While reactive metabolites may function as haptens, the parent drug may also elicit an autoimmune reaction with the production of anti-drug antibodies. Usually, during immunoallergic DILI IgG levels are increased and auto-antibodies against liver-specific and non-liver-specific antigens are detected. Possibly, the observed reduction of IGHG1 in AILI susceptibility is due to glutathione depletion in lymphocytes as suggested by Spielberg and Gordon [32].

### Inflammation and coagulation homeostasis in drug-induced liver injury

The complex interplay between drug-induced inflammation and coagulation homeostasis is the subject of intense research. Synthesized in the liver, plasminogen is converted into plasmin by plasminogen activators and bound to fibrin. Plasmin dissolves fibrin of blood clots and acts as a proteolytic factor in a variety of biological processes, including inflammation. It is activated by the urokinase-type plasminogen activator, collagenases and several complement zymogens, such as C1 and C5. It is inactivated by alpha-2-antiplasmin immediately after dissociation from the clot. Importantly, plasminogen serum concentration was significantly reduced in AILI-susceptible patients after 7 days of APAP treatment. Several reports describe a role of plasminogen in macrophage accumulation during liver repair [33]; plasminogen appears to be essential for neutrophils to accumulate at the border of damaged liver tissue to facilitate repair events [34]. Treatment of mice with APAP caused activation of the coagulation system and protease-activated receptor signaling to contribute to liver injury [35]. Furthermore, fibrin deposition aggravated liver injury; however, plasminogen deficiency reduced APAP liver injury [36] as observed in plasminogen-deficient mice and wild-type mice treated with tranexamic acid, an inhibitor of plasminogen activation. In the present study serum plasminogen concentrations of AILI responders were statistically significantly reduced, therefore demonstrating clinical relevance of the animal findings. Equally, serum plasminogen concentrations were reduced in APAP-treated non-ALT responders, reinforcing the notion that plasminogen is involved in the fine-tuning of liver regeneration after AILI.

Significant down-regulation of kininogen-1 in serum of ALT responders was another finding. This protein functions as a thiol protease inhibitor. High molecular weight kininogen (HMWK) plays an important role in blood coagulation and inflammation and functions as a precursor of kinin, which is part of the vasoactive kinin-kallikrein system. Kininogens also inhibit the thrombin- and plasmin-induced aggregation of thrombocytes and are therefore antithrombotic proteins. Specifically, kininogen is a mediator of inflammation and causes increased vascular permeability, stimulation of nociceptors, release of mediators of inflammation (that is, prostaglandins) and excerts cardioprotective effects (directly via bradykinin action, indirectly via endothelium-derived relaxing factor action).

The complex interplay between collagen, high molecular weight kininogen (HMWK), prekallikrein and factor 12 (Hagemann factor) results in a primary scar. Initially synthesized in the liver, prekallikrein is converted to kallikrein by factor 12 and eventually cleaves HMWK to release bradykinin. In a study by Cordova and colleagues [37] the activities of the Hagemann factor, HMWK and prekallikrein were reduced and dependent on the degree of liver failure. Equally, plasma kallikrein clearance was markedly reduced in APAP intoxicated rat livers [38]. The observed reduction of HMWK in DILI responders is likely the result of APAP poisoning of hepatocytes.

### Regulation of serum apolipoproteins in drug-induced liver injury

The emerging role of lipoproteins in inflammation and cellular signaling is the subject of intense research [39]. Serum proteome profiling of healthy volunteers revealed a significant three-fold down-regulation of ApoA-I in ALT responders after repeated APAP treatment. This protein participates in the reverse transport of cholesterol from tissues to the liver, thereby promoting cholesterol efflux. It functions as a cofactor for the lecithin cholesterol acyltransferase and is a major protein of plasma HDL, typically found in chylomicrons.

ApoA1 expression was negatively correlated with total bilirubin in a patient cohort diagnosed with DILI [23]. These investigators also observed significant regulation of ApoA2, A4 and A4 precursor in NAFLD and various degrees of liver fibrosis. In contrast, ApoC-II was significantly up-regulated in albumin-depleted sera of ALT responders. This protein is part of the very low density lipoprotein (VLDL) particle. The association of ApoC2 with plasma chylomicrons, VLDL and HDL is reversible and functions in the secretion and catabolism of triglyceride-rich lipoproteins. Levels of the related ApoC-III were likewise significantly changed in patients diagnosed with DILI as were ApoC1 levels in serum of patients diagnosed with NAFLD [20].

Furthermore, ApoE expression was more than four-fold up-regulated in DILI responders upon APAP treatment. This lipoprotein mediates binding, internalization and catabolism of lipoprotein particles and serves as a ligand for the ApoE-recognizing receptor of hepatic tissues. Its expression was reported as significantly up-regulated in serum of patients diagnosed with DILI but not in those with non-alcoholic steatohepatitis [20,23]. Early work had shown transcriptional and posttranscriptional regulation of ApoE, ApoA1 and ApoA2 in liver tissue at various pathophysiological states [40] and changes in lipoprotein binding and uptake by hepatocytes during rat liver regeneration [41]. In a proteomic investigation of drug-induced steatosis a moderate but statistically significant up-regulation of ApoE was observed in rat liver, further documenting its prognostic value in predicting DILI. Additionally, DeKroon and co-workers reported an anti-apoptotic role of ApoE [42], while others reported that ApoE protects against severe liver disease in hepatitis C virus infection [43].

Conversely, ApoH was significantly down-regulated in ALT responders after APAP treatment. This protein is synthesized by the liver and secreted in plasma, associates with heparin and dextran sulfate and may prevent activation of the intrinsic blood coagulation cascade by binding to phospholipids on the surface of damaged cells. ApoH was also significantly regulated in rat serum after APAP exposure [24].

Deoxyribonuclease-2-alpha protein was uniquely down-regulated in response to APAP treatment. This protein hydrolyzes DNA with preference for double-stranded DNA and plays a major role in the degradation of nuclear DNA in cellular apoptosis during development. Its lysosomal localization and its known role in the degradation of exogenous DNA encountered by phagocytosis suggest an interplay of this protein with Kupffer cells in AILI. The reduced expression of this protein may be part of the fine tuning of liver regeneration in response to APAP treatment.

Finally, in DILI responders sulfide:quinone oxidoreductase was significantly regulated. This mitochondrial enzyme was shown to catalyze the oxidation of hydrogen sulfide and belongs to the SQRD family of proteins [44]. It is tempting to speculate that this protein is involved in the metabolism of glutathione (GSH) or cysteine conjugates of APAP metabolites.

The following caveats need to be considered. First, the study is based on APAP given to healthy individuals and although robust statistical relationships were determined, further studies are required so that the findings can be extrapolated to other types of DILI in terms of molecular mechanisms and clinical features. Second, the drugs tested in hepatocyte cultures are idiosyncratic agents; nonetheless, the mechanisms of liver injury may differ amongst them. While transcript regulation of serum acute phase reactants could be demonstrated for a wide range of drugs and by comparison with published serum proteome profiling in DILI patients, additional studies are needed to confirm the results obtained after APAP treatment.

## Conclusions

Several serum biomarkers for AILI were identified that may be of utility for other drugs with risk for DILI, thus providing opportunities for drug development programs to improve patient safety.

## Abbreviations

A1AT: Alpha-1 antiproteinase; ADA: Adenosine deaminase; AILI: Acetaminophen-induced liver injury; ALF: Acute liver failure; ALT: Alanine transaminase; APAP: Acetaminophen; Apo: Apolipoprotein; CBB: Coomassie Brilliant Blue; CRP: C-reactive protein; DILI: Drug-induced liver injury; HMWK: High molecular weight kininogen; IEF: Isoelectric focusing; IGHG1: Immunoglobulin gamma-1 chain C region; IL: Interleukin; IPG: Immobilized pH gradient; MALDI: Matrix-assisted Desorption/ionization; NAFLD: Non-alcoholic fatty liver disease; RBP: Retinol binding protein; SAA: Serum amyloid A; TNF: Tumor necrosis factor; TOF: Time of flight.

## Competing interests

The authors declare that they have no competing interests.

## Authors’ contributions

JB and PW designed the study. PW was responsible for the clinical study. BC carried out the experiments. JB and BC reported the experimental data. JB interpreted the data. KL helped with the statistical analysis of the data. JB wrote the manuscript. All authors approved the manuscript.

## Supplementary Material

Additional file 1: Table S1 Details of study participants. Click here for file

Additional file 2: Figure S1 CRP measurements determined in the saliva of healthy volunteers. Click here for file

Additional file 3: Figure S2 A detailed description of the workflow, including protein extraction, two-dimensional gel electrophoresis, image processing, spot-cutting, trypsin in-gel digestion and MALDI-TOF/TOF mass spectrometry of proteins. Click here for file

Additional file 4: Figure S3 Correlation of CRP serum concentrations and ALT activities in APAP-treated healthy individuals. Click here for file

Additional file 5: Table S2 Regulated serum proteins identified in healthy volunteers, in DILI and in non-alcoholic steatohepatitis patients. Click here for file

Additional file 6: Table S3 Drugs used for the treatment of human hepatocyte cultures. Click here for file

Additional file 7: Figure S4 Expression of serum hemopexin in ALT responders, in non-responders and in placebo study subjects. Click here for file
