# Supplementary material for: Serum acute phase reactants hallmark healthy individuals at risk for acetaminophen-induced liver injury
Source: Genome Med. 2013 Sep 27;5(9):86. doi: 10.1186/gm493 (PMC3979026; doi:10.1186/gm493)
Supplement: Additional file 1: Table S1 — Details of study participants. [file gm493-S1.doc]

**Additional file 5: Table S1.** Details of study participants.

| **gender** | **average age** | **age range** | **average weight** | **weight range** |
| --- | --- | --- | --- | --- |
| 21 males | 33,80 | 18 - 55 | 79,78 | 61,7 - 99,4 kg |
| 15 female | 33,00 | 19 - 58 | 68,94 | 49,5 - 91,7 kg |
