# Supplementary figures and images for: Serum acute phase reactants hallmark healthy individuals at risk for acetaminophen-induced liver injury
Source: Genome Med. 2013 Sep 27;5(9):86. doi: 10.1186/gm493 (PMC3979026; doi:10.1186/gm493)

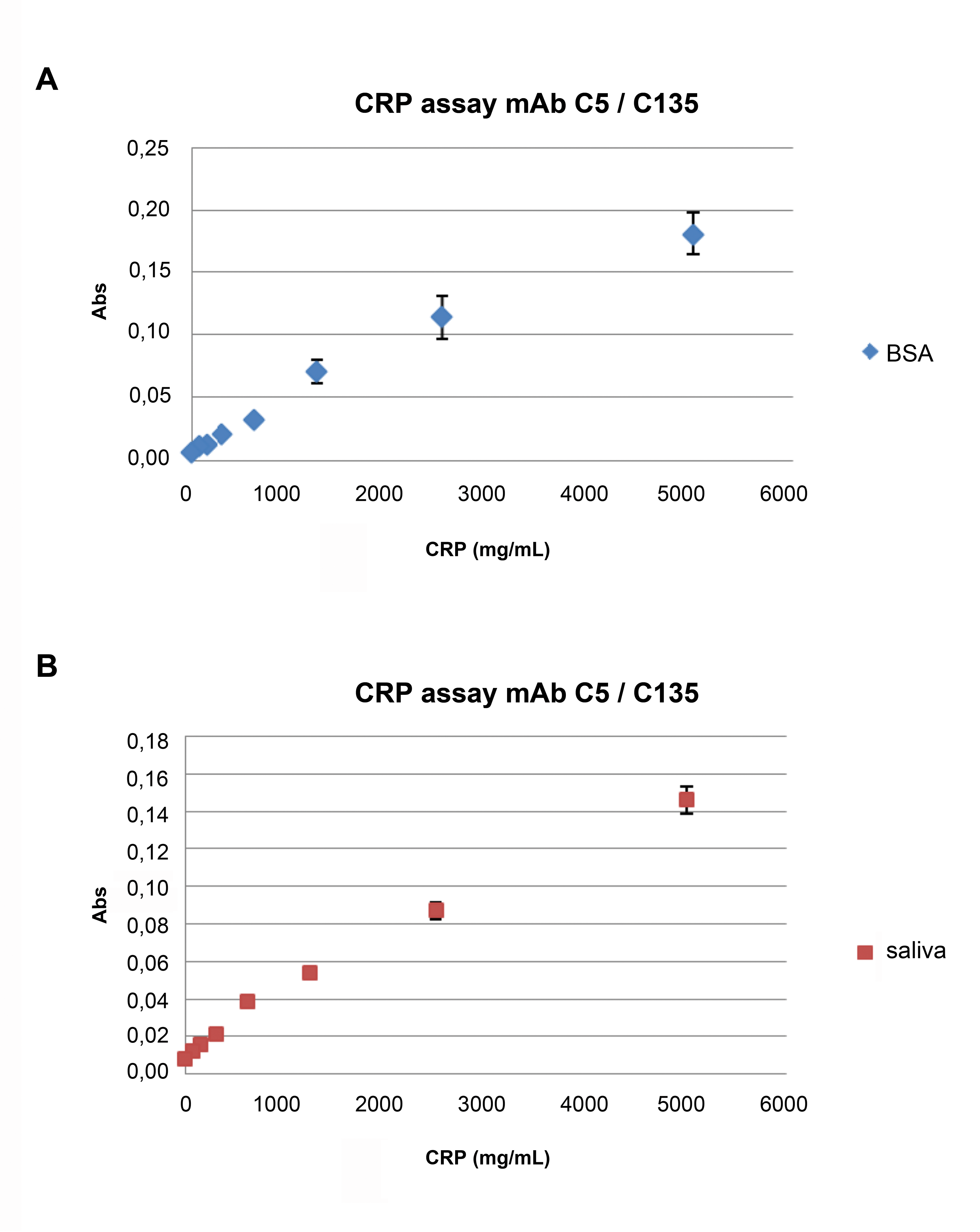

Supplement: Additional file 2: Figure S1 — CRP measurements determined in the saliva of healthy volunteers. [file gm493-S2.tiff]

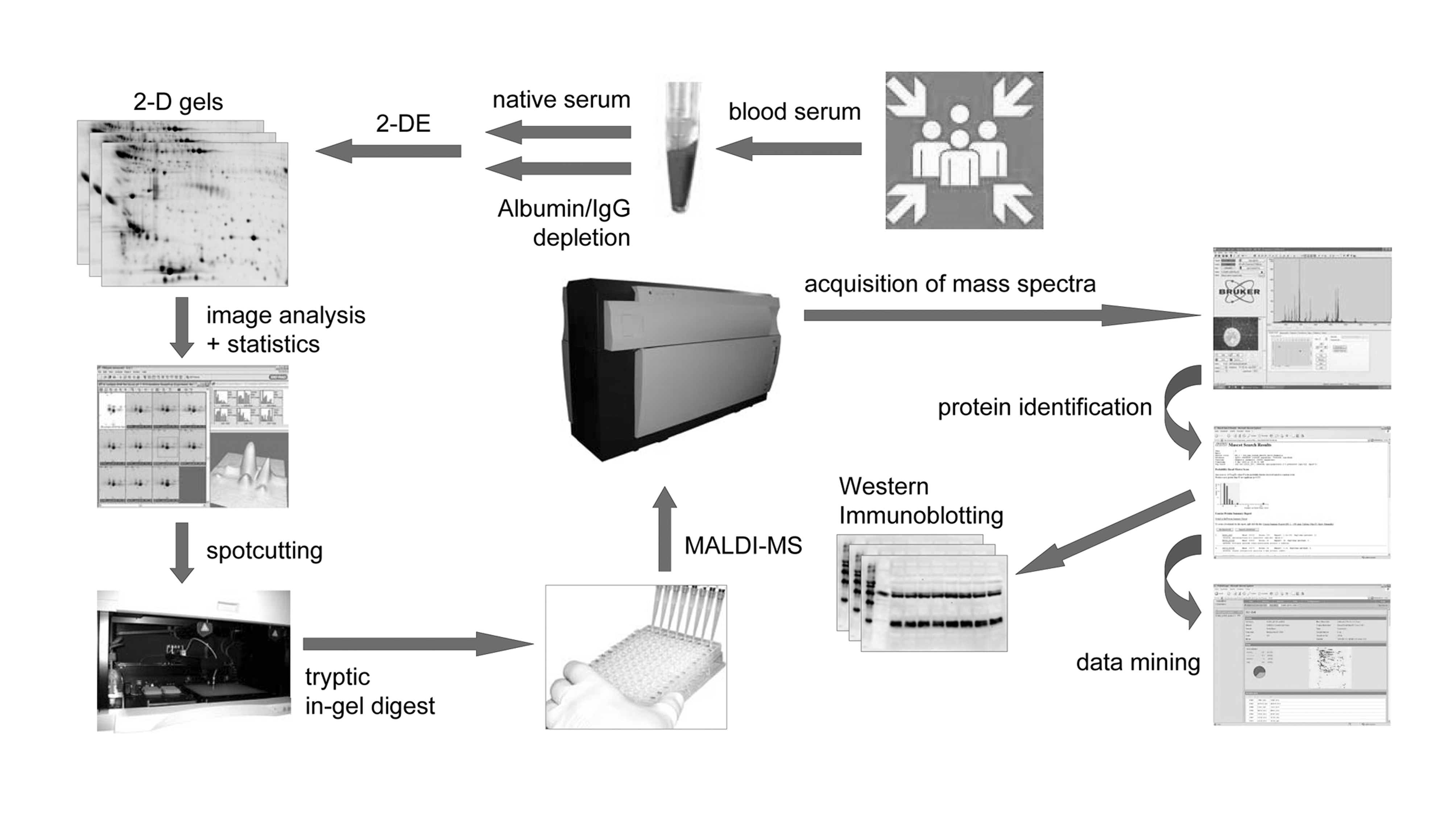

Supplement: Additional file 3: Figure S2 — A detailed description of the workflow, including protein extraction, two-dimensional gel electrophoresis, image processing, spot-cutting, trypsin in-gel digestion and MALDI-TOF/TOF mass spectrometry of proteins. [file gm493-S3.tiff]

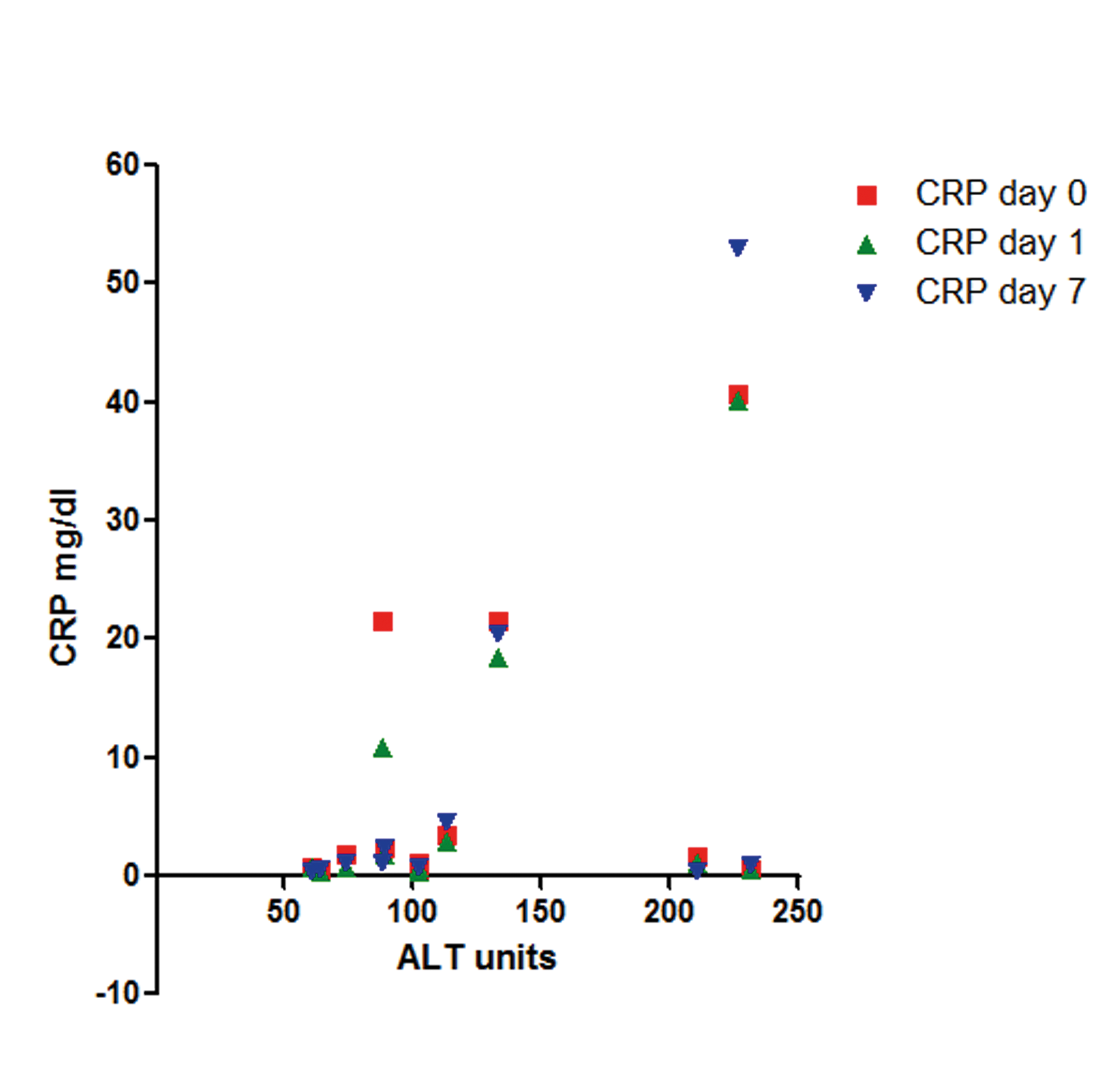

Supplement: Additional file 4: Figure S3 — Correlation of CRP serum concentrations and ALT activities in APAP-treated healthy individuals. [file gm493-S4.tiff]

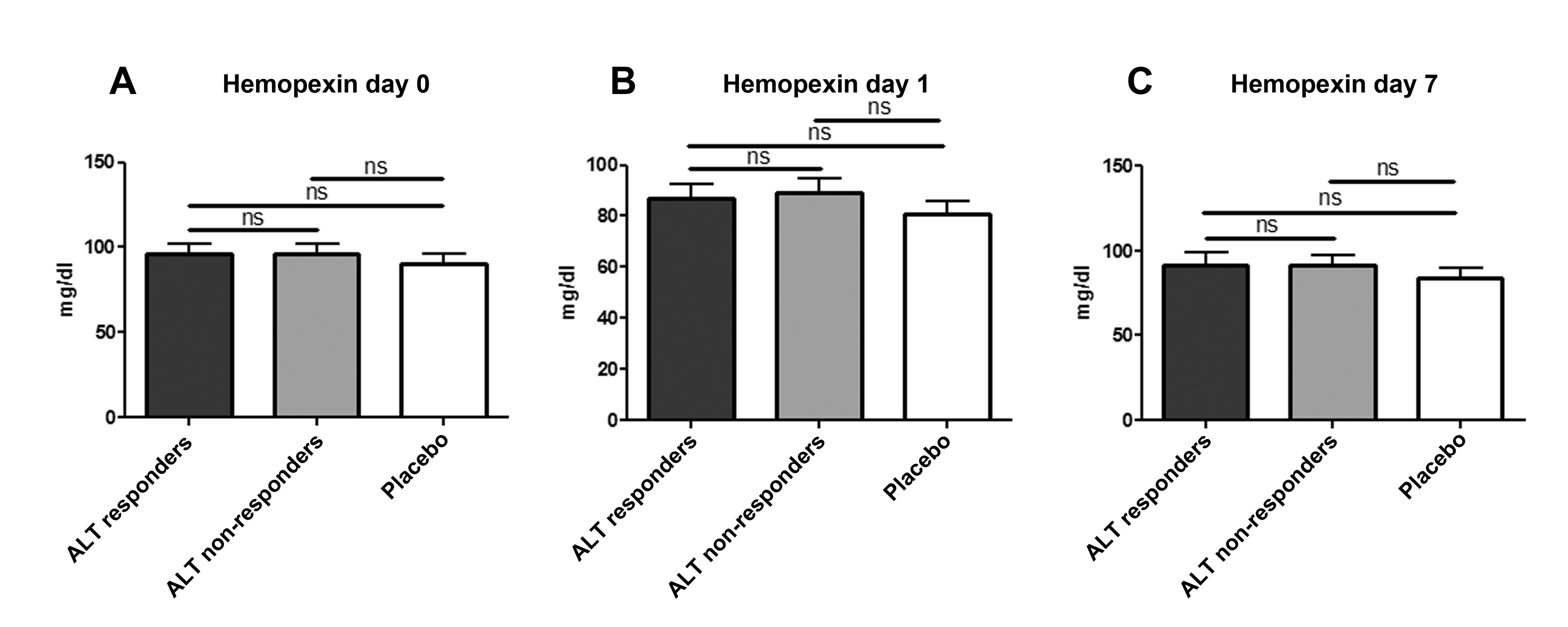

Supplement: Additional file 7: Figure S4 — Expression of serum hemopexin in ALT responders, in non-responders and in placebo study subjects. [file gm493-S7.tiff]
