# Supplementary material for: Serum acute phase reactants hallmark healthy individuals at risk for acetaminophen-induced liver injury
Source: Genome Med. 2013 Sep 27;5(9):86. doi: 10.1186/gm493 (PMC3979026; doi:10.1186/gm493)
Supplement: Additional file 5: Table S2 — Regulated serum proteins identified in healthy volunteers, in DILI and in non-alcoholic steatohepatitis patients. [file gm493-S5.doc]

**Additional file 6: Table S2.** Regulated serum proteins identified in healthy volunteers, in DILI and in NASH patients.

| **Regulated serum proteins** | **Regulated in Borlak et al. study** | **Regulated in DILI study (21)** | **Regulated in NASH study (18)** |
| --- | --- | --- | --- |
| Adenosine deaminase | yes | no | no |
| Alpha1- antiproteinase (A1AT) | yes | yes | no |
| Apolipoprotein A1 | yes | yes | no |
| Beta-2-glycoprotein 1 (apolipoprotein H) | yes | no | no |
| Haptoglobin | yes | yes | no |
| Ig gamma-1 chain C region | yes | no | no |
| Plasminogen | yes | yes | yes |
| Retinol binding protein 4 | yes | yes | yes |
| Sulfide quinone oxidoreductase (SQRDL) | yes | no | no |
| Kininogen-1 | yes | no | no |
| Uncharacterized protein KIAA1688 | yes | no | no |
| Apolipoprotein A4 | yes | yes | yes |
| Apolipoprotein C2 | yes | no | no |
| Apolipoprotein E | yes | yes | no |
| Deoxyribonuclease-2-alpha | yes | no | no |
| Hemopexin | yes | yes | no |
| Hepatoglobin related protein | yes | yes | no |
| Serum amyloid A4 | yes | no | no |
| Ceruloplasmin | yes | no | no |
| Transthyretin | yes | yes | yes |
| α-1-acid glycoprotein 2 | no | yes | yes |
| α-1-acid glycoprotein 1 | no | yes | yes |
| complement component C7 | no | yes | yes |
| serum amyloid P component | no | yes | yes |
| α-2-macroglobulin | no | yes | yes |
| CD5 antigen-like | no | yes | yes |
| C-reactive protein (isoform 1) | no | yes | yes |
| N-acetylmuramoyl-L-alanine amidase (isoform 1) | no | yes | yes |
| fibrinogen γ chain (isoform γB) | no | yes | yes |
| platelet factor 4 | no | yes | yes |
| platelet factor 4 variant | no | yes | yes |
| histidine-rich glycoprotein | no | yes | yes |
| von Willebrand factor | no | yes | yes |
| fibrinogen α chain (isoform 1) | no | yes | yes |
| extracellular matrix protein 1 | no | yes | yes |
| α-2-glycoprotein 1 (zinc) | no | yes | yes |
| gelsolin (isoform 1) | no | yes | yes |
| apolipoprotein C3 | no | yes | yes |
| apolipoprotein A2 | no | yes | yes |
| apolipoprotein A4 precursor | no | yes | yes |
| actin (cytoplasmic) | no | yes | yes |
| Paraoxonase 1 | no | yes | yes |
| complement C4A | no | no | yes |
| complement component 4B (preprotein) | no | no | yes |
| complement component 4A | no | no | yes |
| complement comp 1q subcomp (β chn precursor) | no | no | yes |
| GUGU (β form) | no | no | yes |
| fetuin-B | no | no | yes |
| complement C1s (subcomponent) | no | no | yes |
| Platelet basic protein | no | no | yes |
| fibrinogen β chain | no | no | yes |
| SERPINC1 | no | no | yes |
| antithrombin III variant | no | no | yes |
| prothrombin (fragment) | no | no | yes |
| SERPINF2 | no | no | yes |
| Transgelin 2 | no | no | yes |
| lumican | no | no | yes |
| proteoglycan 4 (isoform A) | no | no | yes |
| Insulin-like growth factor acid labile subunit | no | no | yes |
| insulin-like growth factor binding protein 2 | no | no | yes |
| insulin-like growth factor binding protein 3 | no | no | yes |
| afamin | no | no | yes |
| Apolipoprotein C1 | no | no | yes |
| apolipoprotein B100 | no | no | yes |
| apolipoprotein L1 (isoform 2) | no | no | yes |
| sterile α motif domain-containing protein 9 (isoform 1) | no | no | yes |
| Poly (A) RNA polymerase mitochondrial (isoform 1) | no | no | yes |
| replication initiation-like protein (isoform 1) | no | no | yes |
| Sulfhydryl oxidase 1 (isoform 1) | no | no | yes |
| Angiotensinogen | no | no | yes |
