# Supplementary material for: Serum acute phase reactants hallmark healthy individuals at risk for acetaminophen-induced liver injury
Source: Genome Med. 2013 Sep 27;5(9):86. doi: 10.1186/gm493 (PMC3979026; doi:10.1186/gm493)
Supplement: Additional file 6: Table S3 — Drugs used for the treatment of human hepatocyte cultures. [file gm493-S6.doc]

**Additional file 7: Table 3:** Drugs used for the treatment of human hepatocyte cultures.

| 1 | aspirin | No-DILI-Concern |
| --- | --- | --- |
| 2 | caffeine | No-DILI-Concern |
| 3 | chloramphenicol | No-DILI-Concern |
| 4 | chlorpheniramine | No-DILI-Concern |
| 5 | hydroxyzine | No-DILI-Concern |
| 6 | metformin | No-DILI-Concern |
| 7 | theophylline | No-DILI-Concern |
| 8 | vitamin A | No-DILI-Concern |
|  |  |  |
|  |  |  |
| 1 | adapin | Less-DILI-Concern |
| 2 | allopurinol | Less-DILI-Concern |
| 3 | amitriptyline | Less-DILI-Concern |
| 4 | azathioprine | Less-DILI-Concern |
| 5 | captopril | Less-DILI-Concern |
| 6 | chlorpromazine | Less-DILI-Concern |
| 7 | chlorpropamide | Less-DILI-Concern |
| 8 | cimetidine | Less-DILI-Concern |
| 9 | clofibrate | Less-DILI-Concern |
| 10 | colchicine | Less-DILI-Concern |
| 11 | cyclophosphamide | Less-DILI-Concern |
| 12 | diazepam | Less-DILI-Concern |
| 13 | disopyramide | Less-DILI-Concern |
| 14 | enalapril | Less-DILI-Concern |
| 15 | ethionamide | Less-DILI-Concern |
| 16 | etoposide | Less-DILI-Concern |
| 17 | famotidine | Less-DILI-Concern |
| 18 | fenofibrate | Less-DILI-Concern |
| 19 | fluphenazine | Less-DILI-Concern |
| 20 | furosemide | Less-DILI-Concern |
| 21 | gemfibrozil | Less-DILI-Concern |
| 22 | glibenclamide | Less-DILI-Concern |
| 23 | griseofulvin | Less-DILI-Concern |
| 24 | haloperidol | Less-DILI-Concern |
| 25 | ibuprofen | Less-DILI-Concern |
| 26 | imipramine | Less-DILI-Concern |
| 27 | indomethacin | Less-DILI-Concern |
| 28 | lomustine | Less-DILI-Concern |
| 29 | mefenamic acid | Less-DILI-Concern |
| 30 | meloxicam | Less-DILI-Concern |
| 31 | methyltestosterone | Less-DILI-Concern |
| 32 | mexiletine | Less-DILI-Concern |
| 33 | naproxen | Less-DILI-Concern |
| 34 | nifedipine | Less-DILI-Concern |
| 35 | omeprazole | Less-DILI-Concern |
| 36 | penicillamine | Less-DILI-Concern |
| 37 | phenobarbital | Less-DILI-Concern |
| 38 | phenytoin | Less-DILI-Concern |
| 39 | promethazine | Less-DILI-Concern |
| 40 | quinidine | Less-DILI-Concern |
| 41 | ranitidine | Less-DILI-Concern |
| 42 | simvastatin | Less-DILI-Concern |
| 43 | tetracycline | Less-DILI-Concern |
| 44 | thioridazine | Less-DILI-Concern |
| 45 | tolbutamide | Less-DILI-Concern |
|  |  |  |
|  |  |  |
| 1 | acarbose | Most-DILI-Concern |
| 2 | acetaminophen | Most-DILI-Concern |
| 3 | acetazolamide | Most-DILI-Concern |
| 4 | amiodarone | Most-DILI-Concern |
| 5 | benzbromarone | Most-DILI-Concern |
| 6 | benziodarone | Most-DILI-Concern |
| 7 | carbamazepine | Most-DILI-Concern |
| 8 | chlormezanone | Most-DILI-Concern |
| 9 | ciprofloxacin | Most-DILI-Concern |
| 10 | clomipramine | Most-DILI-Concern |
| 11 | danazol | Most-DILI-Concern |
| 12 | dantrolene | Most-DILI-Concern |
| 13 | diclofenac | Most-DILI-Concern |
| 14 | diltiazem | Most-DILI-Concern |
| 15 | disulfiram | Most-DILI-Concern |
| 16 | erythromycin ethylsuccinate | Most-DILI-Concern |
| 17 | ethambutol | Most-DILI-Concern |
| 18 | flutamide | Most-DILI-Concern |
| 19 | iproniazid | Most-DILI-Concern |
| 20 | ketoconazole | Most-DILI-Concern |
| 21 | labetalol | Most-DILI-Concern |
| 22 | methimazole | Most-DILI-Concern |
| 23 | methyldopa | Most-DILI-Concern |
| 24 | moxisylyte | Most-DILI-Concern |
| 25 | nicotinic acid | Most-DILI-Concern |
| 26 | nimesulide | Most-DILI-Concern |
| 27 | nitrofurantoin | Most-DILI-Concern |
| 28 | papaverine | Most-DILI-Concern |
| 29 | pemoline | Most-DILI-Concern |
| 30 | perhexiline | Most-DILI-Concern |
| 31 | propylthiouracil | Most-DILI-Concern |
| 32 | rifampicin | Most-DILI-Concern |
| 33 | sulfasalazine | Most-DILI-Concern |
| 34 | sulindac | Most-DILI-Concern |
| 35 | tacrine | Most-DILI-Concern |
| 36 | tamoxifen | Most-DILI-Concern |
| 37 | terbinafine | Most-DILI-Concern |
| 38 | ticlopidine | Most-DILI-Concern |
| 39 | valproic acid | Most-DILI-Concern |
